# Thyroid Hormones—An Underestimated Player in Dilated Cardiomyopathy?

**DOI:** 10.3390/jcm10163618

**Published:** 2021-08-16

**Authors:** Karolina Zawadzka, Radosław Dziedzic, Andrzej Surdacki, Bernadeta Chyrchel

**Affiliations:** 1 Students’ Scientific Group at the Second Department of Cardiology, Jagiellonian University Medical College, 2 Jakubowskiego Street, 30-688 Cracow, Poland; karolina.zawadzka@onet.pl (K.Z.); radoslawjozefdziedzic@gmail.com (R.D.); 2 Second Department of Cardiology, Faculty of Medicine, Jagiellonian University Medical College, 2 Jakubowskiego Street, 30-688 Cracow, Poland; surdacki.andreas@gmx.net

**Keywords:** dilated cardiomyopathy, thyroid hormones, thyroid status, prognosis, treatment

## Abstract

Dilated cardiomyopathy (DCM) is the most prevalent cardiomyopathy, typified by left ventricular dilation and systolic dysfunction. Many patients with DCM have altered thyroid status, especially lower levels of free triiodothyronine (T3) and elevated levels of thyroid-stimulating hormone. Moreover, growing evidence indicates that even subtle changes in thyroid status (especially low T3) are linked with a worse long-term prognosis and a higher risk of mortality. Notably, recent discoveries have shown that not only local myocardial thyroid hormones (THs) bioavailability could be diminished due to impaired expression of the activating deiodinase, but virtually all genes involved in TH biosynthesis are also expressed in the myocardium of DCM patients. Importantly, some studies have suggested beneficial effects of TH therapy in patients suffering from DCM. Our aim was to discuss new insights into the association between TH status and prognosis in DCM, abnormal expression of genes involved in the myocardial synthesis of TH in DCM, and the potential for TH use in the future treatment of DCM.

## 1. Introduction

Dilated cardiomyopathy (DCM), the most common form of cardiomyopathy, can be characterized as a nonischemic heart muscle disease with a structural and functional myocardial alteration. DCM is typified by the dilation of the left ventricle or both ventricles, myocyte degeneration, and systolic dysfunction in the absence of cardiovascular diseases such as hypertension, valvular disease, coronary artery disease, or congenital heart disease [1,2]. The etiology of DCM is heterogeneous and encompasses infections, alcohol, drugs, neuromuscular diseases, metabolic dysfunction, autoimmune disorders, and idiopathic causes [3]. The course of DCM may be complicated by heart failure or sudden cardiac death [4].

**Figure 1:**
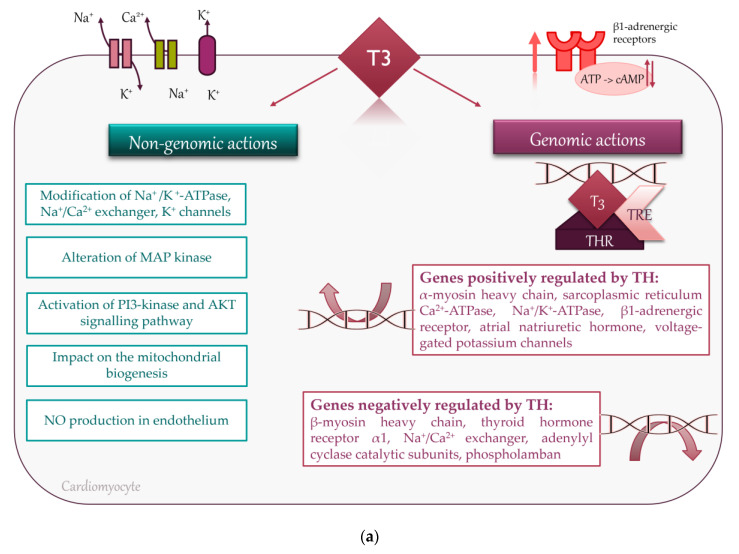

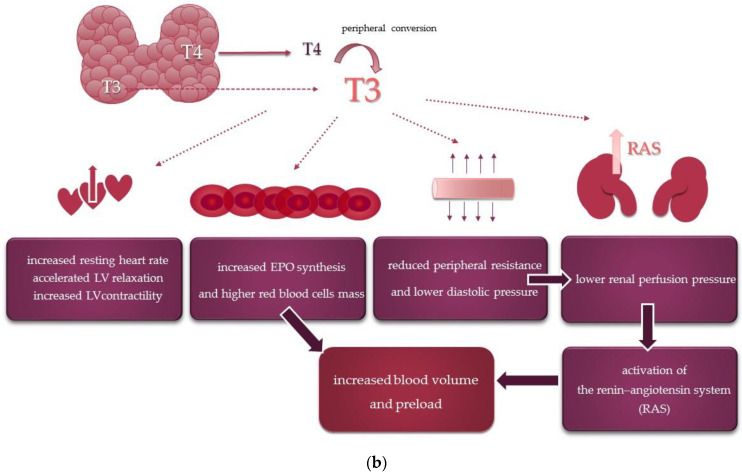
Effects of TH on the cardiovascular system at the cellular level and at organ level: (**a**) Cellular genomic mechanisms include T3 binding to thyroid hormone receptor (THR) in the nucleus of cardiomyocytes, which regulates transcription of various genes by binding to thyroid hormone response elements (TREs). Nongenomic cellular pathways involve modulation of ion channels in the cell membrane of cardiomyocytes, Na^+^/K^+^-ATPase, Na^+^/Ca^2+^ exchanger, MAP kinase, PI3-kinase, and AKT-dependent signaling pathways, mitochondriogenesis and endothelial NO generation; (**b**) TH modulate cardiac inotropy, lusitropy and chronotropy, systemic vascular resistance and preload with a consequent increase in cardiac output. TH—thyroid hormones, T3—triiodothyronine, THR—thyroid hormone receptor, TREs—thyroid hormone response elements, SERCA—sarcoplasmic reticulum calcium-activated ATPase, MAP kinase—mitogen-activated protein kinase, PI3-kinase—phosphatidylinositol 3-kinase, AKT—protein kinase B, NO—nitric oxide, EPO—erythropoietin, LV—left ventricular.

Thyroid hormones (THs) have direct and indirect effects on cardiac function. These effects are mediated by both genomic and nongenomic mechanisms. Triiodothyronine (T3) binds to a specific nuclear receptor, which regulates the transcription of various genes responsible for proper cardiovascular function. Genomic cellular effects of THs are triggered by T3, which binds to specific thyroid hormone receptors (THRs) in the nucleus. THRs are able to regulate transcription via binding to thyroid hormone response elements (TREs). TREs are located within promoters of genes and control the transcription of target genes. Increased transcription of the myosin heavy chain α gene and at the same time, inhibiting the myosin heavy chain β gene, results in an increased rate of contraction. Additionally, increased transcription of sarcoplasmic reticulum calcium-activated ATPase (SERCA), decreased expression of its inhibitory cofactor phospholamban, and enhanced phospholamban phosphorylation, accelerate the rate of both contraction and active relaxation via potentiated sarcoplasmic Ca^2+^ release and uptake. TH also upregulates β-adrenergic receptors, mediating positive chronotropic, inotropic, and lusitropic effects, although these responses can be mitigated by decreased expression of adenylyl cyclase catalytic subunits. Importantly, THs enhance the expression of the hyperpolarization-activated cyclic nucleotide-gated channel gene, governing the sinoatrial node pacing rate. Nongenomic cellular mechanisms of TH action include modulation of ion channels in the cell membrane of cardiomyocytes, Na^+^/K^+^-ATPase, Na^+^/Ca^2+^ exchanger, as well as mitochondriogenesis, nuclear translocation of mitogen-activated protein kinase (MAP kinase) responsible for angiogenesis, activation of phosphatidylinositol 3-kinase (PI3-kinase), and protein kinase B (AKT)-dependent signaling pathways, along with the increased endothelial generation of nitric oxide [5] (Figure 1a).

Beyond metabolic vasodilation linked to higher basal metabolic rate and tissue oxygen demand, THs directly affect cardiovascular hemodynamics by a variety of mechanisms that jointly contribute to an increased cardiac output [5] (Figure 1b).

Recent evidence has made it clear that alterations of TH metabolism are common in DCM and both clinical, and experimental studies have shown a remarkable negative impact of thyroid dysfunction on the prognosis of these patients [6,7,8].

Yet, to the best of our knowledge, data on the comprehensive role of TH abnormalities, including molecular mechanisms underlying local alterations of the expression of genes involved in TH biosynthesis, as well as a possible impact of TH administration on the course of DCM, have not been summarized thus far.

Therefore, our aim was first to gather current knowledge on the influence of abnormal thyroid status on the course of DCM; secondly, to emphasize a potential pathogenetic relevance of altered myocardial expression of genes involved in TH metabolism in DCM, and lastly, to highlight a possible role of TH in future DCM treatment.

## 2. Thyroid Status and Prognosis in DCM

DCM patients with thyroid dysfunction have a worse long-term prognosis [6,7,8,9,10,11,12]. Seminal investigations of the role of TH in the course of DCM found that although TH levels remained within the normal range in 111 DCM patients, the concentrations of free triiodothyronine (FT3) levels were lower and free thyroxine (FT4) higher, compared to 30 age-matched healthy controls [6]. That study also aimed to identify relations between TH and echocardiographic parameters in DCM. Indeed, FT3 or FT3/FT4 ratio correlated negatively with left atrial diameter, right heart chambers size, isovolumetric relaxation time, peak early mitral flow velocity, and severity of mitral regurgitation, while respective relations were negative for left ventricular (LV) ejection fraction, fractional shortening and early mitral flow deceleration time [6]. Additionally, thyroid-stimulating hormone (TSH) and FT4 levels were unrelated to almost any of the echocardiographic parameters, which suggests that FT3 might be a crucial hormone that can affect heart function [6]. Furthermore, low FT3/FT4 ratio, but not FT4 or TSH, was associated with a higher risk of adverse cardiovascular events over about 1 year, mostly (15 out of 16) death from cardiac causes, including heart failure death and sudden arrhythmic death, and was the only independent predictor of adverse outcome upon multivariate adjustment by Cox regression [6].

In a single-center cohort study by Li et al. [7], DCM patients with either subclinical hyperthyroidism or subclinical hypothyroidism, representing 7.1% and 8.2% of the study group, respectively, had higher all-cause mortality rates. During a mean follow-up period of 3.5 years, 218 of 963 patients died, and there was a significant difference in the mortality rate between patients with euthyroidism (21%), subclinical hyperthyroidism (38.2%), and subclinical hypothyroidism (26.6%) [7]. In multivariate analysis, only subclinical hyperthyroidism independently predicted all-cause mortality [7]. Likewise, subclinical hypothyroidism was more frequent than subclinical hyperthyroidism (9% vs. 7%) in a study of 572 DCM patients by Wang et al. [8], while low-T3 syndrome and overt hypothyroidism were found in 4% and 3% of the study subjects, respectively. Notably, lower FT3 and higher TSH were associated with a more advanced stage of heart failure (NYHA III-IV vs. I-II). Importantly, both subclinical and overt hypothyroidism, as well as low T3 syndrome, were independent predictors of all-cause and cardiac death [8], in agreement with the concept of a contribution to thyroid dysfunction to progressive cardiac dysfunction and adverse outcome. However, subclinical hyperthyroidism was not an independent predictor of death [8], in contrast to the report by Li et al. [7]. Notwithstanding, the authors suggested that the latter observation could be biased by the frequent use of β-blockers, which could attenuate detrimental effects of hyperthyroidism, such as increased heart rate or adrenergic overstimulation [8].

In a recent study, Zhao et al. [9] clearly demonstrated that low FT3 was linked to the increased mortality rate in patients with DCM. Moreover, the highest risk was associated with the lowest concentration of FT3, which retained significance after multivariate adjustment.

In a large cohort study, Kannan et al. [10] focused on the impact of thyroid status in 1365 patients with heart failure, whose majority had had cardiomyopathy of nonischemic origin (71%). Most patients were euthyroid, but 14% had low T3 syndrome, 5% subclinical hypothyroidism, 5% subclinical hyperthyroidism, 1% overt hyperthyroidism, and less than 1% overt hypothyroidism. In general, associations between TH and heart failure were consistent with previous reports. A more severe course of heart failure was associated with a higher level of TSH, lower T3, and higher FT4 [10]. The authors also found a significant difference between thyroid status and a composite endpoint encompassing total mortality, cardiac transplantation, or ventricular assist device placement with a higher risk in subclinical hypothyroidism or low T3 syndrome [10].

Wang et al. [11] studied the correlation between thyroid function and a variety of myocardial disorders in 71 DCM patients using multimodal imaging, including cardiac magnetic resonance imaging (MRI), single-photon emission computed tomography (SPECT), and positron emission tomography (PET). Myocardial fibrosis was detected using MRI by late gadolinium enhancement (LGE). Notably, FT3 was the predictor of perfusion and metabolism abnormalities measured by radionuclide tests in both univariate and multivariate analysis [11]. Additionally, they observed a strong correlation between a low level of FT3 and the presence of LGE in DCM. The simultaneous prevalence of LGE and low FT3 was the strongest predictor of all-cause death over a median follow-up of 46 months [11]. Similar findings were reported by Zhang et al. [12] after the extension of the previously described group [11] into 220 DCM subjects: the combination of low FT3 and LGE predicted mortality over about 61 months.

## 3. Hypothyroidism and the Healthy Heart

Beyond the abovementioned reports on the influence of TH deficiency in DCM, a hypothyroid state affects the cardiovascular system also in the absence of cardiac disease via a corollary of pathways. Hypothyroidism is associated with increased systemic vascular resistance (even up to 50%) and endothelial dysfunction, which accounts for mild hypertension in about 30% of patients, affecting mainly diastolic blood pressure with consequent narrowing of pulse pressure [13]. Additionally, as TH also influence the coronary vascular tone and arteriolar angiogenesis, long-term hypothyroidism was associated with decreased coronary blood flow and loss of coronary arterioles in animal studies [13].

As regards direct cardiac effects, beyond bradycardia and pericardial effusion, long-term hypothyroidism may lead to slowed active diastolic relaxation of cardiomyocytes and subtle systolic dysfunction owing to the influence of T3 on myosin isoforms and calcium-binding proteins [13]. Impaired LV relaxation prolongs isovolumic relaxation time and early mitral flow deceleration time with concomitant reduction of the ratio of the peak early and atrial mitral inflow velocity (E/A), all of which are consistent with mild LV diastolic dysfunction [14,15]. Discrete systolic dysfunction, if present, is reflected by prolonged isovolumic contraction [15] and lower LV performance along the longitudinal axis detected by novel echocardiographic techniques [14,15,16], while ejection fraction is generally within the normal range [14,15,16]. Accordingly, although cardiac output is reduced mainly due to bradycardia with a rather minor contribution of direct myocardial effects, hypothyroidism is rarely the primary cause of DCM [13]. Nevertheless, abnormal TH levels may coexist with various causes of DCM, so the exclusion of TH deficiency or excess by means of thyroid function tests is strongly recommended by the current clinical practice guidelines, being a part of the routine diagnostic workup in patients with unexplained DCM [13]. Interestingly, there are case reports that describe patients with DCM and hypothyroidism in whom heart failure symptoms improved after replacement therapy [13,17].

## 4. Changes in Myocardial Expression of Genes Involved in Thyroid Hormone Metabolism in DCM

The thyroid gland produces two tyrosine-based hormones—T4 and T3. The predominant form of circulating TH is T4, which is converted to the bioactive T3 within cells by two different iodothyronines “activating” deiodinases: type 1 deiodinase (D1) or type 2 deiodinase (D2). On the contrary, type 3 deiodinase (D3) inactivates T4 to reverse T3 and T3 to 3,3-diiodothyronine (T2), thereby blocking TH biological effects on a cell-specific basis [18].

**Figure 2:**
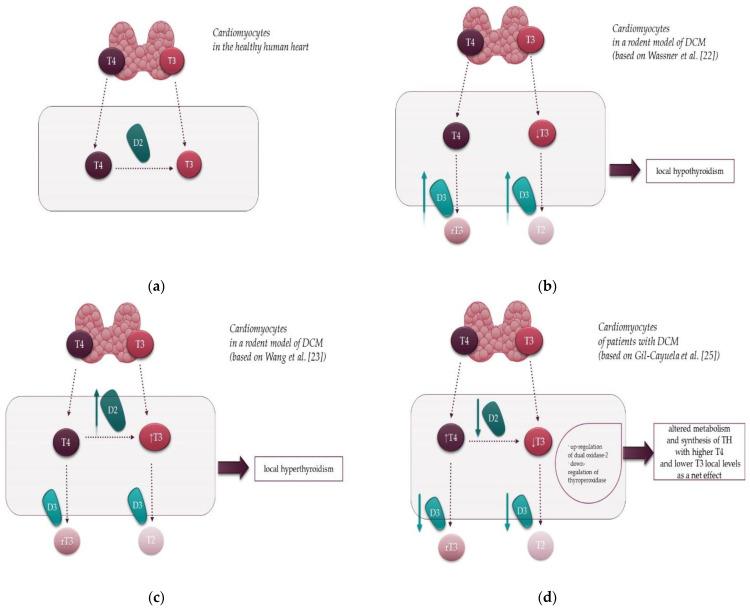
Local effects of type 2 (D2) and type 3 (D3) deiodinases in the normal heart and in DCM: (**a**) The physiological role of deiodinases in the human heart. In the healthy heart, D2 activates T4 to the bioactive hormone T3. D3 expression is undetectable in the healthy heart, except for embryonic development; (**b**) Deiodinase function in cardiomyocytes in a rodent model of DCM by Wassner et al. [22]. In this model inactivating D3 is overexpressed, which leads to local hypothyroidism by inactivating T3 to T2; (**c**) Deiodinase function in a rodent model of DCM by Wang et al. [23]. In this model activating D2 is overexpressed, which leads to local hyperthyroidism by activating T4 to T3; (**d**) Deiodinase function in the human cardiomyocytes in DCM and altered genes involved in TH biosynthesis in patients with DCM according to Gil-Cayuela et al. [25]. In DCM the activity of both D2 and D3 is reduced. Additionally, DCM subjects have increased T4 and decreased T3 levels in the myocardium compared to the control group. Notably, in DCM the myocardial expression of 2 of ll genes involved in TH biosynthesis, i.e., dual oxidase-2 and thyroperoxidase, is upregulated and downregulated, respectively, which may also contribute to a net effect of DCM on cardiac TH levels. T4—thyroxine, T3—triiodothyronine, rT3—reverse T3, T2—3,3-diiodothyronine, D2—type 2 deiodinase, D3—type 3 deiodinase, TH—thyroid hormones, DCM—dilated cardiomyopathy.

Most circulating T3 is derived from the conversion of T4 to T3 by the actions of D1, which is localized in the plasma membrane and expressed mainly in the thyroid and kidneys. Nonetheless, serum TH levels may not accurately reflect their tissue levels as the activating deiodinase (D2) can locally alter TH signaling in a tissue-specific fashion. It is worth noting that although D1 activity is the major source of total T3 in plasma, D2 plays a crucial role in transforming T4 to T3 in extra-thyroidal tissues (Figure 2a).

The posttranslational regulation of D2 expression and its short half-life enables precise control of intracellular generation and concentration of T3 [19]. Importantly, D2 activity is a source of cellular T3 in various tissues, including the human heart. In contrast to D2, D3 expression appears to play a crucial role during embryogenesis and is undetectable in the postnatal healthy heart [20,21]. However, cardiac D3 re-expression was described in some pathological conditions including myocardial infarction, LV pressure overload and some models of heart failure, leading to a state of local hypothyroidism [20,21].

Animal studies tried to delineate the role of D2 and D3 expression in DCM and their effects on cardiac function by changing TH signaling [22,23]. However, the results are inconclusive, demonstrating either local hypothyroid [22] or hyperthyroid [23] state in the myocardium of distinct murine models of DCM. In a study by Wassner et al. [22], there was a 15-fold increase in cardiac-inactivating D3 activity in a transgenic murine model of DCM. D3 overexpression was noted anatomically in cardiomyocytes, thereby contributing to local hypothyroidism in addition to decreased serum T3 and T4 levels (Figure 2b). Moreover, D3 induction was associated with the altered expression of cardiac TH-responsive genes [22]. Specifically, there was a considerable decrease in SERCA-2a mRNA, positively regulated by TH, and a marked increase in the expression of the β-myosin heavy chain, negatively regulated by T3 [22].

In contrast, Wang et al. [23] showed increased D2 activity, leading to a local hyperthyroid state within the myocardium despite unchanged serum T3 levels (Figure 2c). Admittedly, D2 mRNA is physiologically expressed in the human myocardium but not in the heart of a healthy rodent [24]. Nevertheless, Wang et al. [23], by means of real-time quantitative reverse transcriptase-polymerase chain reaction analyses, showed that D2 was overexpressed in the heart of DCM mice. Moreover, it was proposed that enhanced local formation of TH in DCM might promote cardiac growth by activating non-nuclear TH signaling pathways involving protein kinase B (also known as Akt) and p38 MAP kinase. Additionally, treatment with the anti-thyroid drug propylthiouracil improved cardiac function in DCM mice, reduced the expression of the abovementioned hypertrophic markers, and prevented cardiac enlargement. Likewise, the use of propylthiouracil was accompanied by an improvement in ejection fraction and extended lifespan of the DCM animals [23]. 

Deiodinases expression in cardiomyocytes in DCM was recently assessed in a human study by Gil-Cayuela et al. [25], who observed elevated T4 and depressed T3 levels in the LV tissue in DCM versus non-diseased controls (Figure 2d). Interestingly, levels of all three types of deiodinases—D1–D3 were diminished by 20 to 30%, although these differences did not reach statistical significance [25]. Therefore, increased T4 in the cardiac tissue in DCM subjects compared to the control group may reflect downregulation of both types of deiodinases in the DCM heart, i.e., activating deiodinases (D1 and D2) and inactivating D3 deiodinase, because T4 is the substrate of all these enzymes. Furthermore, the study established a new field of research by demonstrating that all genes involved in TH biosynthesis are expressed in the myocardium of patients with DCM [25]. It is worth noting that a comparison of DCM and control samples showed an upregulation of dual oxidase-2 [25] (Figure 2d), a prominent source of hydrogen peroxide which oxidizes iodide into iodine in the thyrocyte [26]. Curiously, myocardial levels of dual oxidase-2 mRNA levels were positively correlated with indices of LV remodeling, reflected by LV diameters and LV mass [25]. In addition, that study also showed a downregulation—albeit unrelated to remodeling parameters—of thyroperoxidase [25] (Figure 2d), an enzyme responsible for the catalysis of iodide oxidation, iodination of tyrosine residues in thyroglobulin, and coupling of iodotyrosines to form iodothyronines attached to thyroglobulin [26].

In addition to local cardiac alteration of genes involved in TH metabolism, another process was proposed as a contributor to locally impaired TH signaling in the DCM myocardium. Modesti et al. [27] suggested that patients with end-stage heart failure due to DCM could exhibit a local hypothyroid state owing to disrupted THRs expression. Moreover, they noted reduced expression of not only THRs(α1 and α2), but also β-adrenergic receptors (β1 and β2) at both gene and protein levels in cardiomyocytes isolated from their end-stage DCM subjects [27]. Importantly, T3 is able to enhance the expression of β1-adrenergic receptors and some of their downstream targets [28,29]. Thus, local cardiac hypothyroidism could potentiate detrimental consequences of myocardial β1-adrenergic receptor downregulation accompanying end-stage heart failure caused by DCM [30], thereby contributing to an adverse outcome.

## 5. Thyroid Hormones as a Possible Treatment for DCM

By a traditional approach, TH is used as a treatment for thyroid dysfunction, and the standard therapy in hypothyroidism is replacement therapy with synthetic L-thyroxine (LT4) [31]. Interestingly, the literature reveals a considerable interest in the usefulness of treatment with TH also in cardiovascular diseases [32]. Beyond the previously mentioned rare cases of DCM linked to hypothyroidism [13,17], some research studies [33,34,35,36,37] claim that TH therapy might be beneficial in the treatment of DCM.

Khalife et al. [33] evaluated the effects of TH treatment in a hamster animal model of DCM associated with subclinical hypothyroidism. They showed that therapeutic doses of TH prevented or attenuated decreases in heart rate or maximum rate of LV pressure increase (dp/dt_max_) and also improved LV ejection fraction. Additionally, TH therapy reduced myocyte loss, normalized impaired coronary blood flow, limited myocardial fibrosis, and alleviated progressive LV dilation.

As early as over 25 years ago, promising results of short-term LT4 supplementation in DCM were reported in euthyroid patients. In a randomized, placebo-controlled study, Moruzzi et al. [34] compared 2 small groups (10 patients each) of euthyroid DCM patients (NYHA functional class II-III). The active treatment group was receiving 100 µg of oral LT4 per day for 7 days, while the control group was taking a placebo. Compared to the controls, patients in the LT4 group had decreased systemic vascular resistance and increased LV ejection fraction and cardiac output at rest [34], as well as better exercise tolerance assessed by a cardiopulmonary exercise test. Furthermore, LT4 supplementation also improved LV contractility, estimated by the relationship between ejection fraction and LV end-systolic wall stress during acute changes in LV afterload [34]. The results were independent of sympathetic activity as hemodynamic responses to dobutamine infusion were unchanged with either LT4 or placebo [34].

These benefits from short-term LT4 supplementation were confirmed by the same research group after the extension of the follow-up until 3 months [35]. In addition to the previous effects observed already after 1 week, reduced LV end-diastolic dimension and increased cardiac output at peak exercise were observed after 3 months of LT4 therapy. Additionally, enhanced hemodynamic responses to dobutamine suggested the ability of a longer LT4 supplementation to up-regulate β1-adrenergic receptor-dependent pathways [35]. It is worth noting that no relevant adverse effects or symptoms of thyroid hyperfunction were observed throughout the treatment period [34,35].

In a more recent study, Badran et al. [36] explored the effect of oral TH treatment on LV mechanics and functional status in patients with DCM using two-dimensional strain imagining, a novel echocardiographic technique. In their study, 60 patients with DCM and no history of thyroid dysfunction were randomized into 2 groups. The first group (40 patients) was taking LT4, and the second one (20 patients) received a placebo for 3 months. At first, patients from the active drug group were taking 25 µg LT4 per day for 4 weeks and then the dose was increased to 50 µg per day for 8 weeks [36]. LT4 therapy significantly improved LV ejection fraction, reduced LV dimensions, decreased LV mass, and reduced the severity of mitral regurgitation in DCM, compared to the placebo group. Interestingly, patients taking LT4 presented an improvement of longitudinal and circumferential LV strain at both segmental and global levels [36]. It is essential to highlight that LT4 therapy was safe and well-tolerated in DCM patients and maintained TH levels within the normal range [36].

Pingitore et al. [37] assessed the short-term effects of synthetic L-triiodothyronine (LT3) treatment over 3 days in 20 patients with systolic heart failure (including 8 patients with DCM) and low-T3 syndrome. A 3-day treatment with LT3 infusions (initial dose 20 µg/m^2^ i.v. adjusted to maintain LT3 levels within the normal range) was well tolerated and quickly ameliorated patients’ hemodynamic parameters in comparison with the control group of 10 heart failure subjects randomized to placebo. In particular, heart rate and LV end-diastolic volume decreased, whereas stroke volume increased in LT3-treated DCM patients [37]. The post-LT3 improvement of cardiac status was not related to higher myocardial oxygen consumption. Concomitantly, neurohormonal hyperactivation was attenuated on i.v. LT3, which was reflected by decreased circulating levels of noradrenaline, N-terminal pro-B Type natriuretic peptide, and aldosterone [37].

Data regarding human randomized controlled studies on TH supplementation in DCM are summarized in Table 1.

The potential usefulness of TH analogs for the treatment of heart dysfunction in DCM opens a new scenario for potential improvement of clinical outcomes in DCM patients. We would like to highlight that no adverse effects were reported on T3/T4 supplementation in DCM patients [34,35,36,37]. However, it must be noted that the study by Badran et al. [36] was single blinded, and the investigators knew which patients were assigned to LT4 or placebo. Other studies did not provide detailed information about blinding, which may have an influence on their credibility or suggest some bias. Moreover, the small number of patients studied, the relatively short period of treatment, as well as differences in the route and dose of TH used, hamper the interpretation of results and limit their implementation in clinical practice.

## 6. Conclusions

A large number of studies suggested that the appropriate function of thyroid glands is relevant in patients with DCM as thyroid dysfunction worsens cardiac function and raises the risk of overall mortality. Considering unfavorable effects of even subclinical TH deficiency on DCM course, the assessment of thyroid status is recommended in every patient with unexplained DCM.

The relationship between low serum concentrations of TH and impaired cardiovascular hemodynamics has provided a rationale for the clinical use of synthetic forms of these hormones beyond well-recognized indications to LT4 supplementation. Short-term treatment of DCM with oral LT4 or intravenous LT3 was associated with beneficial hemodynamic effects without relevant adverse effects. Nevertheless, long-term placebo-controlled studies are necessary to establish the safety and efficacy of long-term TH therapy in further randomized placebo-controlled studies.

In addition, abnormal myocardial expression of genes involved in TH metabolism and impaired cellular TH signaling may contribute to a local hypothyroid state in the myocardium of DCM patients. Whether increased understanding of mechanisms governing cardiac TH-related pathways may contribute to novel therapeutic approaches in DCM remains to be investigated.

## Figures and Tables

**Table 1:** Summary of human-randomized, controlled studies on T3/T4 supplementation in DCM [34,35,36,37].

Publication	Year	Patients *	Treatment	Main Benefits in Patients Randomized to TH Compared to Placebo
Moruzzi et al. [34]	1994	10/10	L-thyroxine (100 µg LT4 per day orally for 7 days), 1 week follow-up	- increased LV ejection fraction
				- higher cardiac output at rest
				- improved LV contractility
				- better exercise tolerance
				- decreased systemic vascular resistance
Moruzzi et al. [35]	1996	10/10	L-thyroxine (100 µg LT4 per day orally for 7 days), 3 months follow-up	- reduced LV end-diastolic dimension
				- increased cardiac output at peak exercise
Badran et al. [36]	2020	40/20	L-thyroxine (25 µg LT4 per day orally for 4 weeks, then 50 µg per day for 8 weeks)	- increased LV ejection fraction
				- reduced LV mass
				- decreased LV dimensions
				- reduced severity of mitral regurgitation
				- improvement of longitudinal and circumferential LV strain
Pingitore et al. [37]	2008	20/10	3-day intravenous infusion of synthetic L-triiodothyronine (initial dose 20 µg/m^2^; adjusted to maintain triiodothyronine levels within the normal range)	- reduced heart rate
				- increased LV stroke volume
				- decreased LV end-diastolic volume
				- alleviated neurohormonal hyperactivation, represented by lower circulating levels of noradrenaline, N-terminal pro-B-type natriuretic peptide and aldosterone

* number of patients on active treatment and placebo, respectively; LT4—L-thyroxine, LV—left ventricular.

## Data Availability

Not applicable.
